# The Ubiquitin Ligase SIAH2 Negatively Regulates Glucocorticoid Receptor Activity and Abundance

**DOI:** 10.3390/biomedicines9010022

**Published:** 2020-12-30

**Authors:** Susan J. Burke, Jessica L. Taylor, Heidi M. Batdorf, Robert C. Noland, David H. Burk, Yongmei Yu, Z. Elizabeth Floyd, J. Jason Collier

**Affiliations:** 1 Laboratory of Immunogenetics, Pennington Biomedical Research Center, Baton Rouge, LA 70808, USA; susan.burke@pbrc.edu; 2 Laboratory of Ubiquitin Biology, Pennington Biomedical Research Center, Baton Rouge, LA 70808, USA; jessica.taylor@pbrc.edu (J.L.T.); yongmei.yu@pbrc.edu (Y.Y.); 3 Laboratory of Islet Biology and Inflammation, Pennington Biomedical Research Center, Baton Rouge, LA 70808, USA; heidi.batdorf@pbrc.edu; 4 Skeletal Muscle Metabolism Laboratory, Pennington Biomedical Research Center, Baton Rouge, LA 70808, USA; robert.noland@pbrc.edu; 5 Cell Biology and Bioimaging Core, Pennington Biomedical Research Center, Baton Rouge, LA 70808, USA; david.burk@pbrc.edu

**Keywords:** adipose tissue, glucocorticoid, inflammation, SIAH2

## Abstract

Glucocorticoids are clinically essential drugs used routinely to control inflammation. However, a host of metabolic side effects manifests upon usage beyond a few days. In the present study, we tested the hypothesis that seven-in-absentia mammalian homolog-2 (SIAH2), a ubiquitin ligase that regulates adipogenesis, is important for controlling adipocyte size, inflammation, and the ability of adipose tissue to expand in response to a glucocorticoid challenge. Using mice with global deletion of SIAH2 exposed or not to corticosterone, we found that adipocytes are larger in response to glucocorticoids in the absence of SIAH2. In addition, SIAH2 regulates glucocorticoid receptor (GR) transcriptional activity and total GR protein abundance. Moreover, these studies reveal that there is an increased expression of genes involved in fibrosis and inflammatory signaling pathways found in white adipose tissue in response to glucocorticoids in the absence of SIAH2. In summary, this is the first study to identify a role for SIAH2 to regulate transcriptional activity and abundance of the GR, which leads to alterations in adipose tissue size and gene expression during in vivo exposure to glucocorticoids.

## 1. Introduction

Glucocorticoids (GCs) are endogenously produced hormones synthesized and secreted from the adrenal glands. The concentrations of GCs are tightly controlled due to their potent effects on both the immune system as well as the metabolic impact of various tissues, such as adipose, liver, pancreas, and skeletal muscle. Supra-physiological increases in GCs, such as would occur in Cushing’s disease and Cushing syndrome, promote adiposity and metabolic disturbances. In addition, GCs are commonly prescribed due to their powerful and beneficial clinical effects to treat a variety of diseases, including allergies, arthritis, autoimmune diseases, and specific cancers [1,2]. However, the impact of chronic GC therapy on body composition in both rodents and humans is drastic, enhances the risk of metabolic diseases, and is not completely understood.

In rodents and humans, there is a clear increase in fat mass and a decrease in lean mass in situations of excess corticosterone (rodent) or cortisol (human) [3,4,5,6]. The increase in fat mass may be a contributor to adverse metabolic events seen in Cushing’s syndrome as well as in patients receiving chronic GC therapy [7,8].Metabolic dysfunction related to increased fat mass is typically accompanied by adipose tissue inflammation. GC-treatment in rodents promotes inflammation in visceral adipose tissue that is strongly associated with glucose intolerance and insulin resistance [9].

One critical regulator of fat mass and adipose tissue inflammation is the ubiquitin ligase seven-in-absentia mammalian homolog-2 (SIAH2) [10,11]. Under conditions of cellular stress, SIAH2 targets multiple proteins for ubiquitin-dependent proteolysis [12]. SIAH2 influences steroid hormone receptor activity in adipose tissue by targeting the nuclear corepressor-1 (NCoR-1) to the proteasome for degradation [13,14,15], leading to agonist-dependent activation of steroid hormone receptors, such as the glucocorticoid receptor [16]. With a high-fat dietary challenge, SIAH2 promotes adipose tissue inflammation during fat mass expansion [11]. These previous findings led us to ask whether SIAH2 activity governs GC-induced changes in adipose tissue inflammation and increased adiposity.

## 2. Experimental Section

### 2.1. Experimental Animals

Wild-type C57BL/6J male mice were purchased from the Jackson Laboratory at 10 weeks of age (Stock # 000664). Siah2^−/−^ (Siah2KO) mice were generated and maintained as described [11,17]. The animals were multi-housed with a 12-h light-dark cycle at 24 °C. Before experimentation, mice had ad libitum access to water and Lab Diet 5015 (Purina Mills, St. Louis, MO, USA). Siah2^+/+^ (wild-type) and Siah2^−/−^ mice were age-matched, and at 12 weeks of age, mice of similar body weight were randomly assigned into study groups. Vehicle control mice were administered 1% ethanol via their drinking water. Corticosterone (Cort; catalog # 27840; Sigma–Aldrich, St. Louis, MO, USA) was reconstituted as described previously to a final concentration of 100 μg/mL in 1% ethanol [4]. Cohort 1 received either Vehicle or Cort in their drinking water for 1 week. Body mass and composition measurements (fat, fat-free, and fluid mass) were generated on study days 0 and 7 using a Bruker Minispec LF110 Time-Domain NMR system. Cohort 2 received either Vehicle or Cort in their drinking water for 3 weeks. During this time, body mass and body composition were determined on days 0 (before administration of Veh and Cort), 8, 16, and 22. Upon completion of both studies, animals were fasted for 4 h, followed by CO_2_ asphyxiation and decapitation. Liver and epididymal white adipose tissue (eWAT) were snap-frozen in liquid nitrogen, and pancreata were fixed in 10% neutral-buffered formalin. Trunk blood was collected, and the serum fraction was separated from whole blood for downstream analysis. All animal experiments were approved by the Pennington Biomedical Research Center Animal Care and Use Committee [IACUC protocols # 947(originally approved in Oct. 2015 and renewed in Sept. 2018) and 1030 (originally approved July 2018)].

### 2.2. Serum ELISA and Liver TG Measurements

Serum insulin was measured using the Mouse Insulin ELISA kit from Mercodia (Uppsala, Sweden). Serum fibroblast growth factor 21 (FGF21) was measured using the Mouse/Rat FGF21 Quantikine ELISA kit (Cat # MF2100) from R&D Systems (Minneapolis, MN, USA). Liver total acyl glycerol measurements have been described in detail previously [18].

### 2.3. Gene Expression Analysis

Total RNA was isolated from homogenized liver and eWAT using Direct-zol RNA Miniprep Kits (Zymo Research) and TriReagent (Molecular Research Center) according to the manufacturer’s instructions. RNA was reverse transcribed to cDNA using the High-Capacity cDNA Reverse Transcription Kit (Applied Biosystems). Real-time PCR was run using SYBR Green or TaqMan (ABI) on the Applied Biosystems 7900HT system. Results were normalized to ubiquitin B (UBB) or hypoxanthine phosphoribosyl transferase 1 (HPRT1), where ΔCT ≤ ±0.5 for the housekeeping gene and data analyzed using the 2^−ΔΔCT^ method with wild-type values used as the calibrator.

### 2.4. Immunohistochemistry and Immunostaining

Pancreatic tissue and eWAT were fixed in 10% neutral buffered formalin for 24 h at 25 °C and subsequently embedded in paraffin. Each tissue was cut into five μm sections. eWAT was stained using hematoxylin and eosin (H&E). Pancreatic tissue was stained for insulin using previously reported procedures [5]. The stained tissue was imaged using NanoZoomer software (Hamamatsu Photonics, Hamamatsu, Japan).

### 2.5. Preparation of Whole-Cell Extracts and Immunoblotting

Adipose tissue was homogenized in a denaturing buffer and processed for immunoblotting as described [11]. Nitrocellulose membranes were incubated with the indicated antibodies for 1–2 h at room temperature or overnight at 4 °C. MemCode staining of the nitrocellulose and β-actin levels were used to confirm equal protein content in each lane. Total protein concentration was determined by bicinchoninic acid assay (BCA) (Pierce).

### 2.6. Transient Transfections

NIH 3T3 fibroblast cells were plated to 50–60% confluence in DMEM (high glucose) containing 10% calf serum and 100 U penicillin/100 μg streptomycin. Transient transfections were performed with one μg of DNA/well and Polyfect (Qiagen) according to the manufacturer’s protocol. The cells were transfected with DNA encoding a GRE driving luciferase gene expression (pGREx3TK-luciferase) and the glucocorticoid receptor alone (control), combined with wild-type hemagglutinin-tagged-Siah2 (HA-Siah2WT) or a dominant-negative RING mutant Siah2 (HA-Siah2H99A-C102A (HA-Siah2 Mut)), as indicated. pcDNA3.1 was used as a negative control and to balance the total amount of DNA in each transfection. Transfection efficiency was monitored using a plasmid expressing GFP expressing under control of the cytomegalovirus promoter [19] as an internal control. All DNA sequences were confirmed using dideoxy sequencing. After forty-eight hours, the medium was exchanged for charcoal-stripped (CS)-DMEM, high glucose, and CS-fetal calf serum. The cells were treated with ethanol (EtOH) as a vehicle control or 100 nM dexamethasone, and whole-cell extracts were harvested 15 h later. For the luciferase reporter assay, the cell lysates were prepared and analyzed for luciferase activity according to the manufacturer’s instructions (Promega) and normalized using total protein content. Glucocorticoid receptor transcriptional activity is reported as the fold change relative light units/milligram of protein relative to glucocorticoid receptor (GR)/GRE with EtOH alone. For Western blot analysis, the cell lysates were prepared by harvesting the cells in nondenaturing buffer (50 mM Tris-Cl, pH 7.4 with 150 mM NaCl, 1 mM EDTA, 1% Triton X-100, 0.5% Igepal) and protease inhibitors (1 μM pepstatin, 1 mM phenylmethylsulfonylfluoride, 50 trypsin inhibitory milliunits aprotinin, and 10 μM leupeptin) followed by sonication and centrifugation at 4 °C × 10 min at 15,700× *g*.

### 2.7. Statistical Analysis

Statistical significance for body weight and body composition was determined using a two-way ANOVA or repeated-measures ANOVA. Statistical significance for all other data was determined using a two-way ANOVA with Tukey’s post-hoc analyses. GraphPad Prism 8.3.0 software (San Diego, CA, USA) was used for statistical analyses. Variability was expressed as the mean with standard deviation or with a standard error of the mean as indicated in the figure legends.

## 3. Results

### 3.1. Corticosterone-Mediated Changes in Body Mass and Body Composition in SIAH2^+/+^ and SIAH2^−/−^ Mice

**Figure 1:**
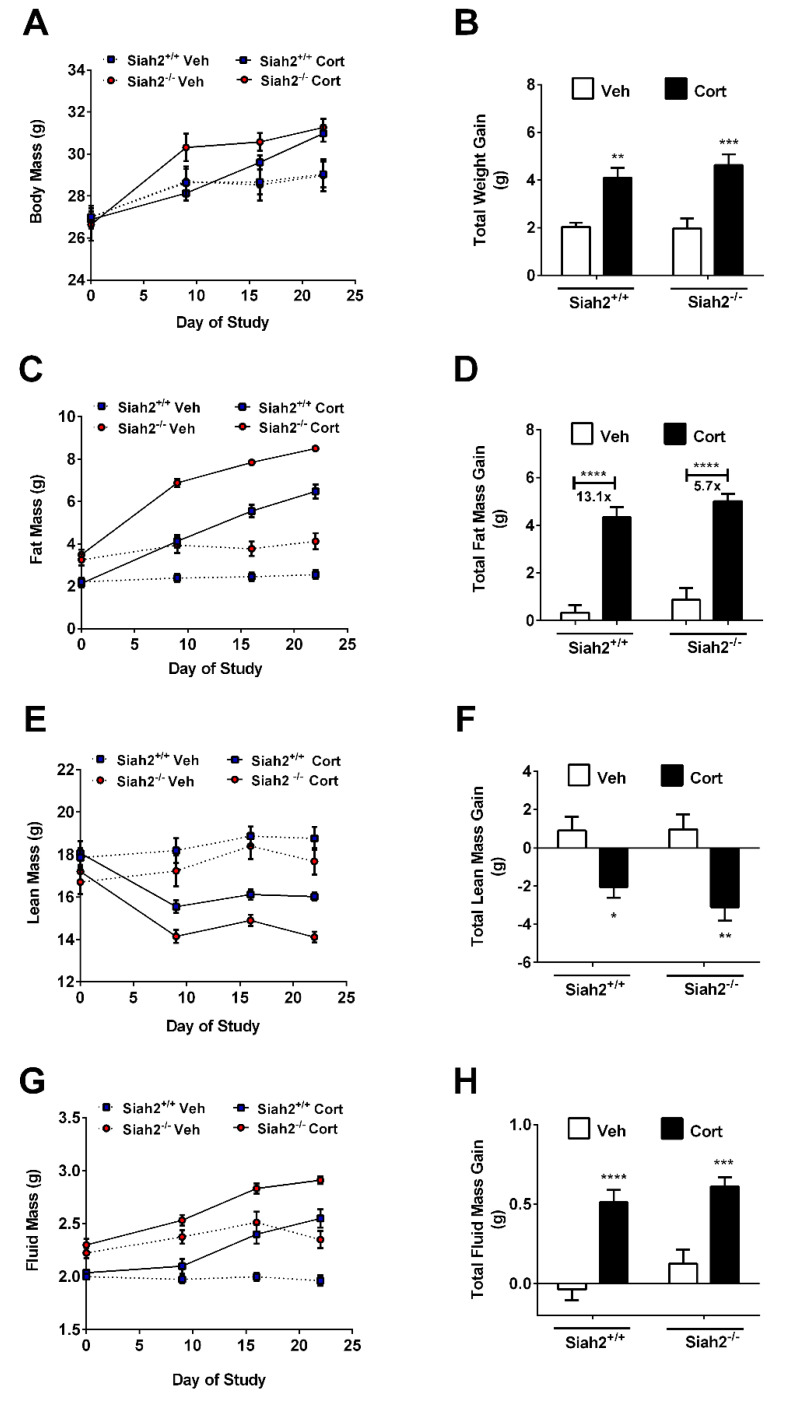
Corticosterone-mediated changes in body mass and body composition in seven-in-absentia mammalian homolog-2 (SIAH2)^+/+^ and SIAH2^−/−^ mice. (**A**) Body mass; (**B**) total weight gain; (**C**) fat mass; (**D**) total fat mass gain; (**E**) lean mass; (**F**) total lean mass change; (**G**) fluid mass; (**H**) total fluid mass change in male SIAH2^+/+^ and SIAH2^−/−^ mice administered either vehicle (Veh) or 100 μg/mL Cort via drinking water for 22 days. *n* = 8–9 per group. Data are represented as means ± SEM. * *p* < 0.05; **, *p* < 0.01; ***, *p* < 0.001; ****, *p* < 0.0001. All asterisks denote within group effects.

**Figure 2:**
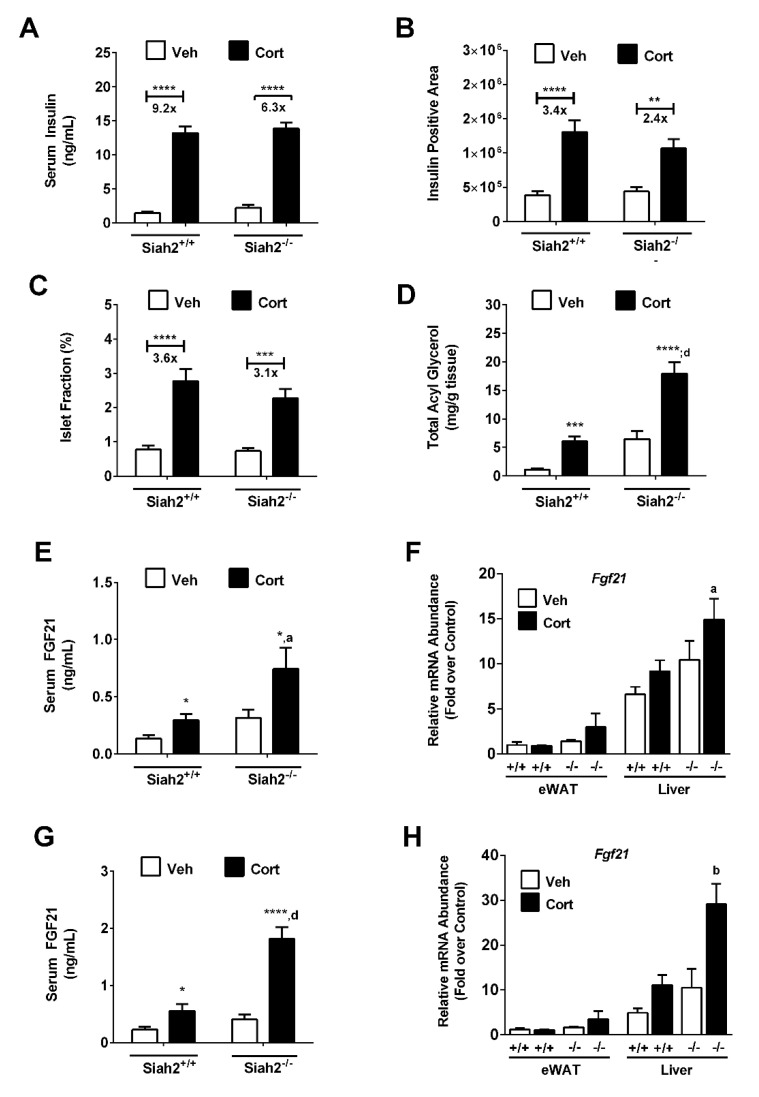
Genetic deletion of SIAH2 alters endocrine responses to Cort and enhances Cort-dependent accumulation of liver triglycerides (TGs). (**A**) Serum insulin; (**B**) insulin positive area; (**C**) islet fraction; and (**D**) liver triglyceride; (**E**) serum FGF21; (**F**) Fgf21 gene expression in epididymal white adipose tissue (eWAT) and liver tissue; (**G**) serum FGF21 and (**H**) Fgf21 gene expression in eWAT and liver tissue. SIAH2^+/+^ and SIAH2^−/−^ mice administered either vehicle (Veh) or 100 μg/mL Cort for 1 week (**E**,**F**) or 22 days (**A**–**D**,**G**,**H**). *n* = 8–9 per group. Data are expressed as means ± SEM. * *p* < 0.05; **, *p* < 0.01; ***, *p* < 0.001; ****, *p* < 0.0001. Asterisks denote within group effects. ^a^, *p* < 0.05; ^b^, *p* < 0.01; ^d^, *p* < 0.0001. Letters denote genotype effects.

Corticosteroid treatment is associated with altered body composition in mice, rats, and humans. Using mice with whole-body deletion of SIAH2 (SIAH2^−/−^), we investigated whether SIAH2 contributed to alterations in fat and lean mass. We observed increased total body mass in response to Cort that was independent of genotype (Figure 1A,B). However, while the increase in fat mass in response to a 22-day exposure to Cort appears similar in SIAH2^−/−^ and control mice, we note two key findings: (1) SIAH2^−/−^ had a larger amount of fat mass at baseline (Figure 1C) and (2) the fold change in fat mass was restricted in the absence of SIAH2 (13.1-fold in control mice versus 5.7-fold in SIAH2^−/−^ mice; Figure 1D). The loss in lean mass was similar in both kinetics and total change between SIAH2^−/−^ and control mice (Figure 1E,F). Finally, the fluid mass was higher in SIAH2^−/−^ at baseline (Figure 1G), which is consistent with their greater fat mass (Figure 1C). Total fluid mass increase in response to Cort was similar in control mice when compared with SIAH2^−/−^ mice (Figure 1H), yet the fold increase in fluid mass is smaller in the absence of SIAH2 compared to control mice.

### 3.2. Genetic Deletion of SIAH2 Alters Endocrine Responses to Cort and Enhances Cort-Dependent Accumulation of Liver Triglycerides (TGs)

Corticosterone exposure promoted insulin resistance leading to hyperinsulinemia in rodents and humans. Loss of SIAH2 did not protect against the development of hyperinsulinemia (Figure 2A). However, the fold increase in circulating insulin levels in response to Cort was reduced in the absence of SIAH2 (9.2-fold in control mice versus 6.3-fold in SIAH2^−/−^ mice; Figure 2A). When analyzing pancreatic tissue, the absolute amount of insulin-positive area and number of islets per section were similar between control and SIAH2^−/−^ mice in Cort treated mice (Figure 2B,C); however, the fold change in these parameters was restricted in the absence of SIAH2 (3.4-fold in control mice versus 2.4-fold in SIAH2^−/−^ mice; Figure 2B and 3.6-fold in control mice versus 3.1-fold in SIAH2^−/−^ mice; Figure 2C). Conversely, in liver tissue, we observed a significant increase in triglycerides both in the baseline and after Cort exposure in SIAH2^−/−^ mice (Figure 2D). We noted that vehicle treated SIAH2^−/−^ mice had as much TG in the liver as control mice given Cort (Figure 2D). In addition, circulating FGF21 was elevated in SIAH2^−/−^ mice in response to one week of Cort exposure (Figure 2E). This finding was consistent with FGF21 gene expression in the liver of SIAH2^−/−^ mice after one week of Cort exposure (Figure 2F). The concentration of FGF21 in serum remained elevated in SIAH2^−/−^ mice after three weeks on the Cort regimen (Figure 2G), again congruent with mRNA accumulation in the liver (Figure 2H). We noted that there was no significant accumulation of FGF21 mRNA in eWAT tissue following Cort administration (Figure 2F,H).

### 3.3. Genetic Deletion of SIAH2 Leads to Dysregulation of Genes Supporting Inflammation in eWAT within One Week

**Figure 3:**
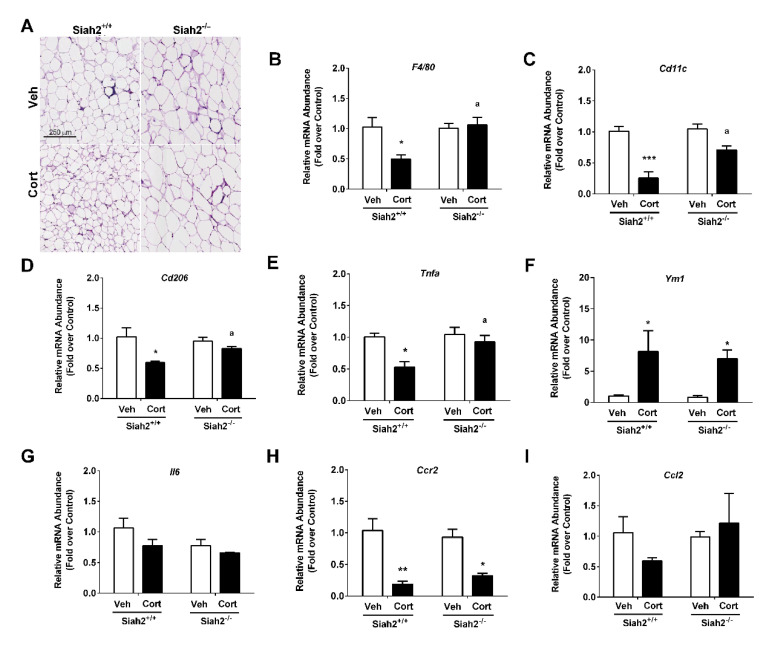
Genetic deletion of SIAH2 leads to dysregulation of genes supporting inflammation in eWAT within one week. (**A**) Hematoxylin and eosin (H&E) staining of epididymal white adipose tissue; mRNA abundance of (**B**) *F4/80*, (**C**) *Cd11c*, (**D**) *Cd206,* (**E**) *Tnfa*, (**F**) *Ym1*, (**G**) *Il6*, (**H**) *Ccr2*, and (**I**) *Ccl2* in SIAH2^+/+^ and SIAH2^−/−^ mice administered either vehicle (Veh) or 100 μg/mL Cort via drinking water for 1 week. Data are expressed as means ± SEM. *, *p* < 0.05; **, *p* < 0.01; ***, *p* < 0.001. Asterisks denote within group effects. ^a^ *p* < 0.05 for the genotype effect. *n* = 6–8 per group.

White adipose tissue expansion occurs via increasing adipogenesis, hypertrophy, or both [20,21,22]. When examining eWAT from mice receiving Cort for 1 week, we observed larger adipocytes in the SIAH2^−/−^ group, while the control mice, expressing SIAH2, displayed reduced size and increased number of adipocytes (Figure 3A)

Gene expression analyses revealed a suppression in F4/80 (aka EMR1) by Cort exposure, which was not present in SIAH2^−/−^ mice (Figure 3B). In addition, the gene encoding *Cd11c* was also suppressed by Cort in SIAH2^+/+^ but was not altered in SIAH2^−/−^ mice (Figure 3C). Both the *Cd206* (Figure 3D) and Tnfa (Figure 3E) genes followed similar patterns. However, other genes, such as *Ym1* (Figure 3F) and *Il6* (Figure 3G), were not affected by either Cort treatment or SIAH2 deletion. We noted that the gene encoding *Ccr2* remained responsive to Cort in both the presence and absence of SIAH2 (Figure 3H), while *Ccl2*, encoding the ligand for CCR2, also did not appear to be impacted by Cort or absence of SIAH2 (Figure 3I). Thus, several genes encoding either immune cell markers, chemokines, and cytokines were differentially regulated by Cort depending on the presence or absence of SIAH2.

### 3.4. Inflammation-Associated Gene Expression Is Modulated by the Presence of SIAH2 during Three Weeks of Drug Regimen

**Figure 4:**
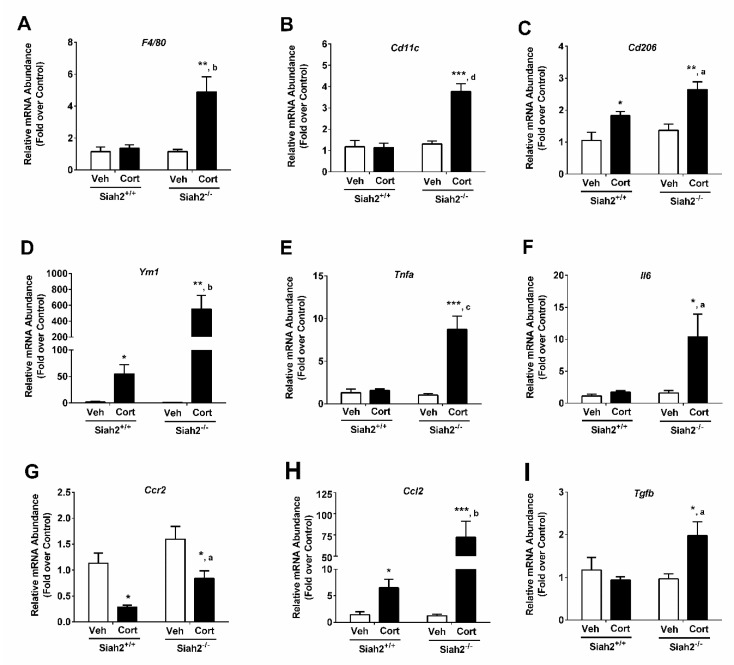
Inflammation-associated gene expression in eWAT was modulated by the presence of SIAH2 during a three week Cort regimen. mRNA abundance of (**A**) *F4/80*, (**B**) *Cd11c*, (**C**) *Cd206*, (**D**) *Ym1*, (**E**) *Tnfa*, (**F**) *Il6*, (**G**) *Ccr2*, (**H**) *Ccl2,* and (**I**) *Tgfb* in SIAH2^+/+^ and SIAH2^−/−^ mice administered either vehicle (Veh) or 100 μg/mL Cort via drinking water for 22 days. Data are expressed as means ± SEM. * *p* < 0.05; ** *p* < 0.01; *** *p* < 0.001. Asterisks denote within group effects. ^a^ *p* < 0.05; ^b^ *p* < 0.01; ^c^ *p*< 0.001; ^d^ *p* < 0.0001. Letters denote genotype effects. *n* = 6/group.

Upon exposure to Cort for three weeks, the gene encoding F4/80 was similar between SIAH2^+/+^ mice receiving vehicle and those receiving Cort (Figure 4A). However, F4/80 expression was elevated in the SIAH2^−/−^ mice exposed to Cort (Figure 4A). F4/80 is a general marker of macrophage content in the adipose tissue along with *Cd11c* as a marker of pro-inflammatory macrophages. As shown in Figure 4B, the expression of the *Cd11c* gene was most highly expressed by exposure to Cort in SIAH2^−/−^ mice. The genes encoding *Cd206* (Figure 4C), *Ym1* (Figure 4D), *Tnfa* (Figure 4E), and *Il6* (Figure 4F) show increased expression with Cort in SIAH2^−/−^ but not SIAH2^+/+^ mice. However, expression of *Ccr2* (Figure 4G) was repressed by Cort, although less efficiently in SIAH2^−/−^ mice. Finally, expression of *Ccl2* (Figure 4H) and *Tgfb* (Figure 4I) was increased by Cort in SIAH2^−/−^ mice. *Cd206* and *Ym1* were included as markers of anti-inflammatory macrophages. In addition, markers related to cytokine/chemokine content in the adipose tissue (*Tnfα, Il6, Tgfβ, Ccl2*, and the CCL2 receptor *Ccr2*) signal the recruitment of macrophages to the adipose tissue. Finally, we noted that the markers we assayed for macrophage content were not expressed in adipocytes. However, *Tnfα, Ccl2, Tgfβ,* and *Il6* were expressed by multiple cell types within adipose tissue as a whole, including adipocytes.

### 3.5. Cort-Dependent Activation of Fibrotic Genes Occurs in the Absence of SIAH2

**Figure 5:**
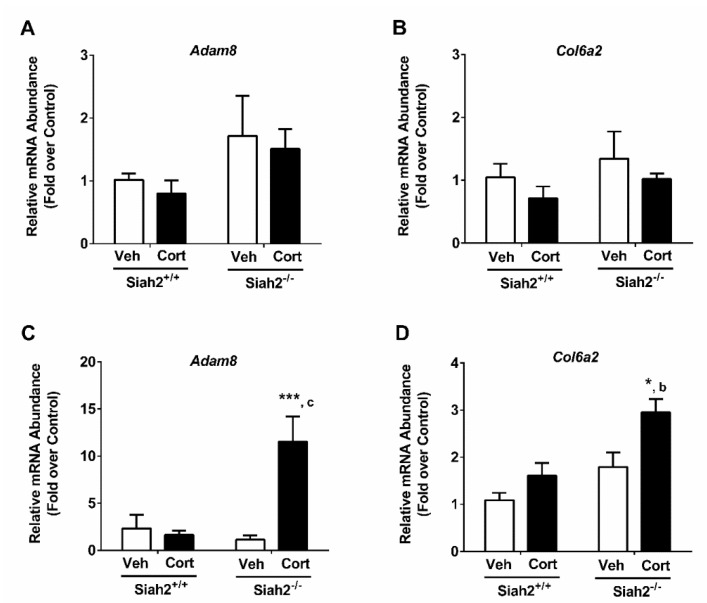
Cort-dependent activation of fibrotic genes occurs in eWAT in the absence of SIAH2. Gene expression of (**A**) *Adam8* and (**B**) *Col6a2* in SIAH2^+/+^ and SIAH2^−/−^ mice administered either vehicle (Veh) or 100 μg/mL Cort via drinking water for 1 week. Transcript levels of (**C**) *Adam8* and (**D**) *Col6a2* in SIAH2^+/+^ and SIAH2^−/−^ mice administered either vehicle (Veh) or 100 μg/mL Cort via drinking water for 22 days. Data are expressed as means ± SEM. *, *p* < 0.05; ***, *p* < 0.001. Asterisks denote within-group effects. ^b^ *p* < 0.01; ^c^ *p* < 0.001. Letters denote genotype effects. *n* = 6/group.

Next, we found that, in the presence or absence of SIAH2, a one-week exposure to Cort does not increase expression of the fibrotic genes *Adam8* (Figure 5A) or *Col6a2* (Figure 5B) in eWAT. Similar results were seen in the presence of SIAH2 when the Cort exposure was increased to 3 weeks (Figure 5C,D). However, when SIAH2 is genetically deleted, there is a significant upregulation of these genes following a 3-week treatment with oral Cort (Figure 5C,D). Taken together with Figure 3 and Figure 4, these data highlight that Cort-dependent modulation of inflammatory and fibrotic gene expression is regulated, at least in part, by SIAH2.

### 3.6. Glucocorticoid-Regulated Genes Are Influenced by Both Cort Exposure and the Presence of SIAH2

**Figure 6:**
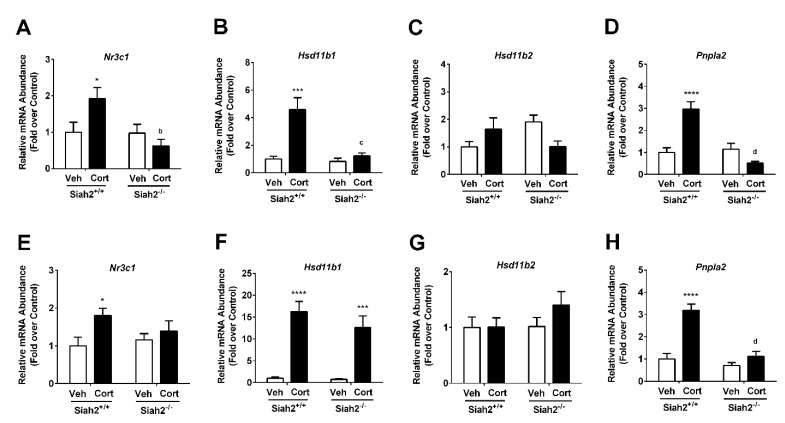
Differential requirement for SIAH2 to sustain expression of glucocorticoid regulated genes in eWAT. Gene expression of (**A**) *Nr3c1*, (**B**) *Hsd11b1*, (**C**) *Hsd11b2*, and (**D**) *Pnpla2* in SIAH2^+/+^ and SIAH2^−/−^ mice administered either vehicle (Veh) or 100 μg/mL Cort via drinking water for one week. Transcript levels of (**E**) Nr3c1, (**F**) Hsd11b1, (**G**) Hsd11b2, and (H) Pnpla2 in SIAH2^+/+^ and SIAH2^−/−^ mice administered either vehicle (Veh) or 100 μg/mL Cort via drinking water for 22 days. Data are expressed as means ± SEM. * *p* < 0.05; *** *p* < 0.001; **** *p* < 0.0001. Asterisks denote within group effects. ^b^ *p* < 0.01; ^c^ *p* < 0.001; ^d^ *p* < 0.0001. Letters denote genotype effects.

The expression of the gene encoding the glucocorticoid receptor (*Nr3c1*) was enhanced in the presence of acute (1 week; Figure 6A) and chronic (3 weeks; Figure 6E) exposure to Cort. The enzyme 11β-hydroxysteroid dehydogenase type-1 (11β-HSD1) catalyzes the conversion of inert 11-keto corticosteroids to active glucocorticoids (e.g., corticosterone); *Hsd11b1*, which encodes the 11β-HSD1 protein, was regulated by glucocorticoid exposure [23]. Expression of *Hsd11b1* was markedly enhanced in response to one week of cort exposure (Figure 6B); this expression was lost in the SIAH2^−/−^ mice at the one week time point. Expression of *Hsd11b1* was sustained during three weeks of expression in SIAH2^+/+^ mice and was restored in SIAH2^−/−^ mice (Figure 6F). By contrast, expression of *Hsd11b2*, which encodes the enzyme that catalyzes the reverse reaction (active corticosteroids to inactive steroids), did not appear to be regulated by either cort or SIAH2 (Figure 6C,G). The GR target gene *Pnpla2* (encoding the protein adipose triglyceride ligase) was increased in expression during both one (Figure 6D) and three weeks (Figure 6H) of cort exposure. Interestingly, SIAH2 was required for this increase in gene expresssion at both time points.

### 3.7. Glucocorticoid Receptor Abundance and Activity Are Modulated by SIAH2

**Figure 7:**
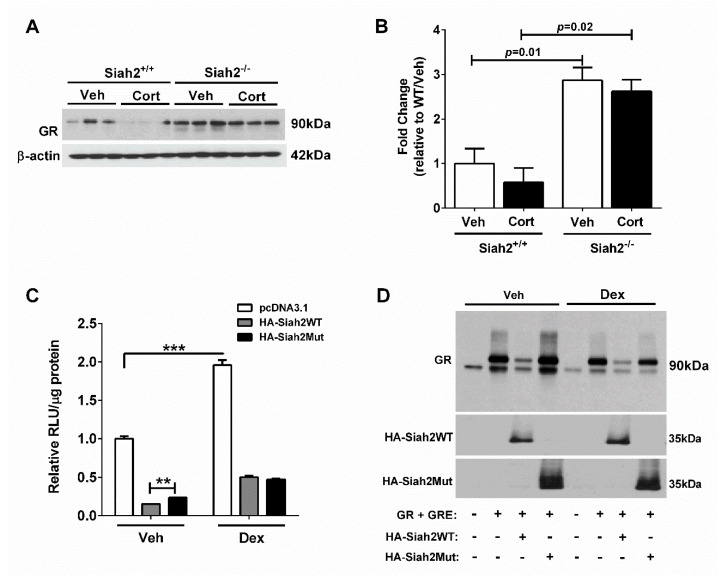
Glucocorticoid receptor abundance and activity are modulated by SIAH2. (**A**) Glucocorticoid receptor (GR) abundance in eWAT of SIAH2^+/+^ and SIAH2^−/−^ mice administered either vehicle or 100 μg/mL Cort via drinking water for one week. (**B**) Densitometric analysis of immunoblot shown in panel (**A**) with Veh indicated by white bars and Cort indicated by the black bars. (**C**) NIH 3T3 cells transfected with the pGREx3TK-luciferase plasmid, GR overexpression plasmid, and either pcDNA3.1, HA-Siah2WT, or HA-Siah2 Mut. 48 h post-transfection cells were stimulated with EtOH (Veh) or Dex (100 nM) for 15 h. (**D**) Cells were treated as described in panel (**C**) and immunoblotted for GR and SIAH2 abundance. Data are represented as means ± SEM. ** *p* < 0.01; *** *p* < 0.001. The data in C and D are representative of experiments that were performed independently four times.

Because several genes in eWAT displayed a clear dependence on SIAH2 for their regulation by Cort (Figure 3, Figure 4 and Figure 5), we investigated GR abundance in both SIAH2^+/+^ and SIAH2^−/−^ mice exposed to Cort for one week. Cort exposure reduced the GR protein by 42% (Figure 7A,B), a known phenotype of GR ligand activity [24]. However, in SIAH2^−/−^ mice, GR abundance was elevated in the absence of ligand and also displayed no downregulation by the presence of Cort (Figure 7A; quantification in Figure 7B). By three weeks of Cort exposure, GR protein levels were reduced in response to ligand but did not show a clear genotype-dependent effect (Supplementary Figure S1). We next examined GR activity using a luciferase-reporter construct in NIH 3T3 fibroblast cells. In this assay, dexamethasone (a synthetic GR agonist) induced a 1.96 fold increase in promoter activity (Figure 7C; white bars). Transfection of plasmids expressing either wild-type Siah2 (HA-Siah2WT; grey bars) or an enzymatically inactive mutant of Siah2 (HA-Siah2 Mut; black bars) revealed that Siah2 reduced promoter-luciferase activity in both the basal state and when the ligand was present (Figure 7C). Furthermore, the turnover of the GR protein was significantly enhanced in response to overexpression of WT Siah2 (Figure 7D). However, unlike ligand activated-GR activity, GR protein stability was dependent on Siah2 enzymatic activity in the presence or absence of a GR ligand (Figure 7D). Taken together, Siah2 regulates GR abundance using its enzymatic function while controlling Dex-dependent GR transcriptional output independently of its known enzymatic activity.

## 4. Discussion

Glucocorticoids are powerful anti-inflammatory drugs that, when administered chronically, are also the most common cause of drug-related metabolic pathologies [8,25]. In this study, we tested the hypothesis that the ubiquitin ligase SIAH2 is important for mediating the impact of glucocorticoids on adipose tissue size, inflammation, and markers of tissue remodeling. Several key findings emerged from this work: (1) adipocytes are larger in response to glucocorticoids in the absence of SIAH2. (2) SIAH2 regulates glucocorticoid receptor transcriptional activity. (3) SIAH2 regulates glucocorticoid receptor abundance. (4) In the absence of SIAH2, there is increased expression of genes involved in fibrosis and inflammatory signaling pathways in white adipose tissue in response to glucocorticoids.

These findings are particularly interesting in light of past work showing that SIAH2 is important for the regulation of adipose tissue size and inflammation in mice fed a high-fat diet [11]. Although Siah2 depletion dampens adipose tissue inflammation and fibrosis when the gonadal fat expands to respond to chronic dietary lipid challenge [11], loss of Siah2 increases inflammation and fibrosis in gonadal fat upon Cort exposure (Figure 3, Figure 4 and Figure 5). Macrophage polarization toward elevated levels of pro-inflammatory markers is particularly evident after chronic exposure to Cort with Siah2 deficiency. Siah2-mediated suppression of GR activity (e.g., Figure 7C) may account for this difference as GR in adipocytes is associated with increased adipose tissue inflammation with glucocorticoid treatment [26]. In addition, there is a correlation between the expression of the genes encoding the GR (i.e., *Nr3c1*) and *Hsd11b1* (encoding the enzyme that converts inactive glucocorticoid to active glucocorticoid) increasing, while pro-inflammatory gene expression is generally suppressed, particularly at the one-week time point of cort exposure (compare Figure 4 and Figure 6). This phenotype also appears to be largely dependent on the presence of SIAH2. 

SIAH2 promotes adipogenesis [10]. Consequently, the increased inflammatory response to Cort with SIAH2 deficiency could possibly be attributed to impaired GR-stimulated adipogenesis, leading to enhanced polarization of pro-inflammatory macrophages during adipose tissue expansion. Clearly, the present and previous work collectively demonstrate the importance of SIAH2 in the regulation of adipose tissue mass and adipocyte size. In addition, the present study is, to our knowledge, the first report of SIAH2 regulating both GR abundance and transcriptional activity. Our data are also in agreement with previous studies documenting the necessity of inflammation to support adipose tissue mass expansion [27]. Thus, it is possible that the compensatory upregulation of inflammatory genes in response to Cort in SIAH2^−/−^ mice reflects the reduced ability of adipose tissue mass to expand when SIAH2 is absent (Figure 4).

The GR, via binding to glucocorticoid hormones, regulates the expression of various genes by numerous molecular mechanisms, including direct effects at the promoter level, competition for cofactors (e.g., squelching), or induction of specific genes that feedback to inhibit certain pathways [28,29]. Here we demonstrated for the first time that the ubiquitin ligase SIAH2 provides regulatory activity towards the GR at two levels: abundance and activity. The GR is known to be ubiquitylated, which controls its degradation [30]. However, not all of the GR is degraded in response to a signal-specific stimulus; residual GR is also regulated by trafficking from the nucleus to the cytoplasm to shut off its transcriptional activity [31]. We found that transcriptional regulation by GR, and its modulation by SIAH2, is a novel control point restraining GR-mediated transcription in adipose tissue. This regulation may also extend to other tissues, although this remains to be described.

An additional interesting and important finding that arose from this study was the observation that lipids in the liver increased in SIAH2^−/−^ mice, which was enhanced when the mice were exposed to glucocorticoids (Figure 2D). These data fit with SIAH2 being required for adipose tissue expansion, and thus, when expansion is limited when SIAH2 is absent, the excess lipid is stored in lean tissue (e.g., liver). The storage of lipid in lean tissue is generally viewed to produce negative outcomes [32,33]. Thus, the role of SIAH2 to support healthy adipose tissue in states of high-fat feeding [10,11] and glucocorticoid exposure (present study) is becoming apparent.

With a growing cadre of selective GR agonists being developed in attempts to reduce or eliminate metabolic side effects while retaining the important anti-inflammatory properties of such drugs [34,35,36,37,38], the GR regulation by SIAH2 is a novel aspect to be considered. Future studies will focus on whether the introduction of selective GR modulating compounds dissociate GR from SIAH2 control. In addition, other considerations include understanding the important role for SIAH2 in adipose tissue, where its regulation of GR activity and abundance fits is now a key part of the biology.

## Data Availability

Data available on request by contacting either of the corresponding authors.

## Supplementary Materials

The following are available online at https://www.mdpi.com/2227-9059/9/1/22/s1, Figure S1: Glucocorticoid receptor abundance is regulated by the presence of corticosterone.

## References

[1] Cain D.W., Cidlowski J.A. (2017). Immune regulation by glucocorticoids. Nat. Rev. Immunol..

[2] Kadmiel M., Cidlowski J.A. (2013). Glucocorticoid receptor signaling in health and disease. Trends Pharmacol. Sci..

[3] Reid I.R., Wattie D.J., Evans M.C., Stapleton J.P. (1996). Testosterone Therapy in Glucocorticoid-Treated Men. Arch. Intern. Med..

[4] Burke S.J., Batdorf H.M., Huang T.-Y., Jackson J.W., Jones K.A., Martin T.M., Rohli K.E., Karlstad M.D., Sparer T.E., Burk D.H. (2019). One week of continuous corticosterone exposure impairs hepatic metabolic flexibility, promotes islet β-cell proliferation, and reduces physical activity in male C57BL/6 J mice. J. Steroid Biochem. Mol. Biol..

[5] Burke S.J., Batdorf H.M., Eder A.E., Karlstad M.D., Burk D.H., Noland R.C., Floyd Z.E., Collier J.J. (2017). Oral Corticosterone Administration Reduces Insulitis but Promotes Insulin Resistance and Hyperglycemia in Male Nonobese Diabetic Mice. Am. J. Pathol..

[6] Freedman M.R., Horwitz B.A., Stern J.S. (1986). Effect of adrenalectomy and glucocorticoid replacement on development of obesity. Am. J. Physiol. Integr. Comp. Physiol..

[7] Peeke P.M., Chrousos G.P. (1995). Hypercortisolism and Obesity. Ann. N. Y. Acad. Sci..

[8] Schäcke H., Döcke W.-D., Asadullah K. (2002). Mechanisms involved in the side effects of glucocorticoids. Pharmacol. Ther..

[9] Do T.T.H., Marie G., Héloïse D., Guillaume D., Marthe M., Fève B., Marion B. (2019). Glucocorticoid-induced insulin resistance is related to macrophage visceral adipose tissue infiltration. J. Steroid Biochem. Mol. Biol..

[10] Kilroy G., Burk D.H., Floyd Z.E. (2016). Siah2 Protein Mediates Early Events in Commitment to an Adipogenic Pathway. J. Biol. Chem..

[11] Kilroy G., Carter L.E., Newman S., Burk D.H., Manuel J., Möller A., Bowtell D.D., Mynatt R.L., Ghosh S., Floyd Z.E. (2015). The ubiquitin ligase Siah2 regulates obesity-induced adipose tissue inflammation. Obesity.

[12] Maneix L., Catic A. (2016). Touch and go: Nuclear proteolysis in the regulation of metabolic genes and cancer. FEBS Lett..

[13] Catic A., Suh C.Y., Hill C.T., Daheron L., Henkel T., Orford K.W., Dombkowski D.M., Liu T., Liu X.S., Scadden D.T. (2013). Genome-wide Map of Nuclear Protein Degradation Shows NCoR1 Turnover as a Key to Mitochondrial Gene Regulation. Cell.

[14] Zhang J., Guenther M.G., Carthew R.W., Lazar M.A. (1998). Proteasomal regulation of nuclear receptor corepressor-mediated repression. Genes Dev..

[15] Perissi V., Aggarwal A., Glass C.K., Rose D.W., Rosenfeld M.G. (2004). A Corepressor/Coactivator Exchange Complex Required for Transcriptional Activation by Nuclear Receptors and Other Regulated Transcription Factors. Cell.

[16] Schulz M., Eggert M., Baniahmad A., Dostert A., Heinzel T., Renkawitz R. (2002). RU486-induced Glucocorticoid Receptor Agonism Is Controlled by the Receptor N Terminus and by Corepressor Binding. J. Biol. Chem..

[17] Frew I.J., Dickins R.A., Cuddihy A.R., Del Rosario M., Reinhard C., O’Connell M.J., Bowtell D.D. (2002). Normal p53 Function in Primary Cells Deficient for Siah Genes. Mol. Cell. Biol..

[18] Burke S.J., Batdorf H.M., Burk D.H., Noland R.C., Eder A.E., Boulos M.S., Karlstad M.D., Collier J.J. (2017). db/db Mice Exhibit Features of Human Type 2 Diabetes That Are Not Present in Weight-Matched C57BL/6J Mice Fed a Western Diet. J. Diabetes Res..

[19] Matsuda T., Cepko C. (2004). Electroporation and RNA interference in the rodent retina in vivo and in vitro. Proc. Natl. Acad. Sci. USA.

[20] Spalding K.L., Arner E., Westermark P.O., Bernard S., Buchholz B.A., Bergmann O., Blomqvist L., Hoffstedt J., Näslund E., Britton T. (2008). Dynamics of fat cell turnover in humans. Nat. Cell Biol..

[21] Knittle J.L., Timmers K., Ginsberg-Fellner F., E Brown R., Katz D.P. (1979). The growth of adipose tissue in children and adolescents. Cross-sectional and longitudinal studies of adipose cell number and size. J. Clin. Investig..

[22] Salans L.B., Knittle J.L., Hirsch J. (1968). The role of adipose cell size and adipose tissue insulin sensitivity in the carbohydrate intolerance of human obesity. J. Clin. Investig..

[23] Sai S., Esteves C.L., Kelly V., Michailidou Z., Anderson K., Coll A.P., Nakagawa Y., Ohzeki T., Seckl J.R., Chapman K.E. (2008). Glucocorticoid regulation of the promoter of 11beta-hydroxysteroid dehydrogenase type 1 is indirect and requires CCAAT/enhancer-binding protein-beta. Mol. Endocrinol..

[24] Bellingham D.L., Sar M., A Cidlowski J. (1992). Ligand-dependent down-regulation of stably transfected human glucocorticoid receptors is associated with the loss of functional glucocorticoid responsiveness. Mol. Endocrinol..

[25] Zhou P.-Z., Zhu Y.-M., Zou G.-H., Sun Y.-X., Xiu X.-L., Huang X., Zhang Q.-H. (2016). Relationship between Glucocorticoids and Insulin Resistance in Healthy Individuals. Med Sci. Monit..

[26] Dalle H., Garcia M., Antoine B., Boehm V., Do T.T.H., Buyse M., Ledent T., Lamazière A., Magnan C., Postic C. (2019). Adipocyte Glucocorticoid Receptor Deficiency Promotes Adipose Tissue Expandability and Improves the Metabolic Profile Under Corticosterone Exposure. Diabetes.

[27] Asterholm I.W., Tao C., Morley T.S., Wang Q.A., Delgado-Lopez F., Wang Z.V., Scherer P.E. (2014). Adipocyte Inflammation Is Essential for Healthy Adipose Tissue Expansion and Remodeling. Cell Metab..

[28] De Bosscher K., Vanden Berghe W., Haegeman G. (2003). The interplay between the glucocorticoid receptor and nuclear factor-kappaB or activator protein-1: Molecular mechanisms for gene repression. Endocr. Rev..

[29] Burke S.J., Goff M.R., Updegraff B.L., Lu D., Brown P.L., Minkin S.C., Biggerstaff J.P., Zhao L., Karlstad M.D., Collier J.J. (2012). Regulation of the CCL2 Gene in Pancreatic beta-Cells by IL-1beta and Glucocorticoids: Role of MKP-1. PLoS ONE.

[30] Wallace A.D., Cidlowski J.A. (2001). Proteasome-mediated Glucocorticoid Receptor Degradation Restricts Transcriptional Signaling by Glucocorticoids. J. Biol. Chem..

[31] Freeman B.C., Yamamoto K.R. (2001). Continuous recycling: A mechanism for modulatory signal transduction. Trends Biochem. Sci..

[32] Unger R.H. (2003). Lipid overload and overflow: Metabolic trauma and the metabolic syndrome. Trends Endocrinol. Metab..

[33] Unger R.H. (2002). Lipotoxic Diseases. Annu. Rev. Med..

[34] Ali A., Thompson C.F., Balkovec J.M., Graham D.W., Hammond M.L., Quraishi N., Tata J.R., Einstein M., Ge L., Harris G. (2004). NovelN-Arylpyrazolo[3,2-c]-Based Ligands for the Glucocorticoid Receptor: Receptor Binding and in Vivo Activity. J. Med. Chem..

[35] Rosen J., Miner J.N. (2005). The Search for Safer Glucocorticoid Receptor Ligands. Endocr. Rev..

[36] Schäcke H., Berger M., Rehwinkel H., Asadullah K. (2007). Selective glucocorticoid receptor agonists (SEGRAs): Novel ligands with an improved therapeutic index. Mol. Cell. Endocrinol..

[37] Weinstein D.S., Gong H., Doweyko A.M., Cunningham M., Habte S., Wang J.H., Holloway D.A., Burke C., Gao L., Guarino V. (2011). Azaxanthene Based Selective Glucocorticoid Receptor Modulators: Design, Synthesis, and Pharmacological Evaluation of (S)-4-(5-(1-((1,3,4-Thiadiazol-2-yl)amino)-2-methyl-1-oxopropan-2-yl)-5H-chromeno[2,3-b]pyridin-2-yl)-2-fluoro-N,N-dimethylbenzamide (BMS-776532) and Its Methylene Homologue (BMS-791826). J. Med. Chem..

[38] Burke S.J., May A.L., Noland R.C., Lu D., Brissova M., Powers A.C., Sherrill E.M., Karlstad M.D., Campagna S.R., Stephens J.M. (2015). Thiobenzothiazole-modified Hydrocortisones Display Anti-inflammatory Activity with Reduced Impact on Islet beta-Cell Function. J. Biol. Chem..

