# Supplementary material for: The Ubiquitin Ligase SIAH2 Negatively Regulates Glucocorticoid Receptor Activity and Abundance
Source: Biomedicines. 2020 Dec 30;9(1):22. doi: 10.3390/biomedicines9010022 (PMC7823448; doi:10.3390/biomedicines9010022)
Supplement: Supplementary file 1 [file biomedicines-09-00022-s001.pdf]

biomedicines

Supplementary Material

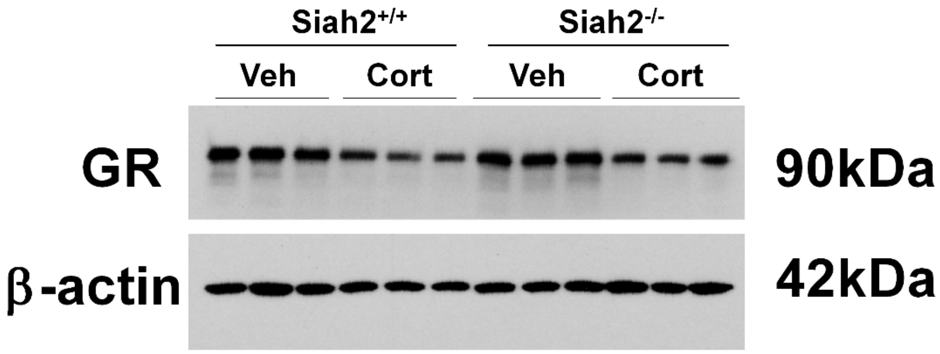

Scheme 1. Glucocorticoid receptor abundance is regulated by the presence of corticosterone. GR abundance in eWAT of SIAH2+/+ and SIAH2-/- mice that were administered either vehicle (Veh) or 100 ug/mL Cort via drinking water for three weeks.
